# The Role of Internship Programs in Fostering School‐to‐Work Transitions in Secondary Schools: Paths for Transversal Skills and Orientation (PCTOs)

**DOI:** 10.1002/jad.12531

**Published:** 2025-07-03

**Authors:** Sara Germani, Mara Marini, Irene Stanzione

**Affiliations:** ^1^ Department of Psychology and Socialization Processes Sapienza University of Rome Rome Italy; ^2^ Current Affiliation: Department of Human Sciences Link Campus University of Rome Rome Italy; ^3^ Department of Neuroscience, Imaging and Clinical Sciences “Gabriele D’ Annunzio” University of Chieti‐Pescara Chieti Italy; ^4^ Department of Dynamic and Clinical Psychology, and Health Studies Sapienza University of Rome Rome Italy

**Keywords:** career preparedness, paths for transversal skills and orientation (PCTOs), school‐to‐work transitions, transversal skills, vocational education

## Abstract

**Introduction:**

The school‐to‐work transition (STWT) represents the initial significant career milestone in young people's lives. In Italy, the Paths for Transversal Skills and Orientation (PCTOs) were introduced in upper secondary schools as an educational tool to support STWT. PCTOs aim to equip students with transversal skills that promote employability and personal development. Given limited research on PCTO effectiveness, this study examines specific PCTO characteristics in enhancing students' transversal skills and career resources.

**Methods:**

A cross‐sectional study was conducted between April and May 2024. 567 students (M age = 17.30, SD age = 1.02, 72% males) from two vocational schools (grades 11–13) in central Italy completed a questionnaire assessing four PCTO indicators: orientation value, student involvement in PCTO design, number of PCTO experiences, and quality of the tutor‐student relationship. Transversal skills and career motivational resources were also evaluated.

**Results:**

A path analysis model revealed positive associations between PCTO indicators and students' transversal skills, which, in turn, were related to career motivational resources. Contrary to expectations, the quality of the tutor–student relationship did not show direct associations with the outcomes. Additionally, results suggested that engaging in various PCTO activities may reduce the clarity of career orientation.

**Conclusions:**

Findings highlight that a quality PCTO planning fosters transversal skills, indirectly enhancing career resources. While different PCTO experiences support skill development, they may reduce the “orientation” value of the Paths. This study underscores the value of PCTOs in preparing young people for the labor market, emphasizing the importance of targeted, skill‐oriented experiences.

## 1 Introduction

The 2030 Agenda (United Nations 2015) for sustainable development identifies achieving inclusive, high‐quality education for all children, adolescents, and adults, especially those in vulnerable conditions, as well as the reduction of economic disparities and the socioeconomic and political inclusion of the population, as key goals for the year 2030. In line with these objectives, the Recommendation on Key Competences for Lifelong Learning (European Commission 2018) highlights the essential role of education in fostering the skills and competencies necessary for young people's personal development, professional success, and integration into the labor market. It is, therefore, within the purview of the EU and its member states to bridge the gap between educational and socioeconomic realities, address the discrepancy between academic and vocational fields, and tackle the issue of NEETs (Not in Education, Employment, or Training). Indeed, while the attainment of upper secondary education is an essential qualification for young people to contribute effectively to societal development, the effectiveness of educational institutions in this regard also depends on their ability to equip young individuals with the skills and competencies necessary for successful integration into the labor market.

The most recent recommendations on pathways to educational success (Council of the European Union 2022) aim to address issues related to student success by improving career guidance, which becomes a valuable tool to support students in developing skills and abilities related to career choice and management. In these recommendations, participation in work‐study programs related to curricular studies is fundamental in significantly improving students' employment prospects. Integrating theory and practice, such programs facilitate the development of technical and interpersonal skills, including the knowledge, abilities, and attitudes essential for effective work performance, while enhancing the likelihood of a successful transition from education to employment (Covacevich 2021; OECD 2024a, 2024b; Vermeire et al. 2022).

In vocational literature, the school‐to‐work transition (STWT)—typically between the end of compulsory education and the attainment of full‐time employment—addresses many long‐standing issues concerning the interrelationships between school, employment, and training (Ryan 2001). The STWT represents the initial significant career milestone in the lives of the majority of young individuals, marking their transition from the role of a student to that of a worker (Masdonati et al. 2022; Presti et al. 2022; Simões 2022). Broadly defined, the STWT begins within the educational system, continues between the end of secondary school and finding employment, and extends into the initial stages of work experience. This broader perspective acknowledges the crucial preparatory role of schools in this process, underscoring the importance of equipping young people with suitable vocational preparation (Nicholson 1990; Vermeire et al. 2022) starting in early adolescence (Marciniak et al. 2021). Indeed, career preparedness is associated with a range of adaptive outcomes at the academic, social, and personal levels (Lent 2013; Marciniak et al. 2022). Despite differing theoretical perspectives (e.g., Lent 2013; Rudolph et al. 2017; Savickas 2020; Chen 2003) and instruments used for measurement (Marciniak et al. 2022), career preparedness has been identified as a significant predictor of professional success among young people (Marciniak et al. 2022).

The complexity of the STWT is linked to the fact that many factors influence career pathways. Particularly, recent research has indicated that STWT is shaped by both individual and contextual factors (Blokker et al. 2023; Masdonati et al. 2022; OECD 2024a; Hirschi 2012). Individual factors encompass skills, abilities, attitudes, and goals that facilitate preparation to enhance job readiness and the probability of attaining quality employment and a competitive starting salary (e.g., Akkermans et al. 2013; Fu et al. 2023; Koen et al. 2012; Presti et al. 2022). On the other hand, contextual factors, such as market conditions, the overall economy, and particularly the educational context, also play a complex role (Blokker et al. 2023). Regarding the educational environment, the structural characteristics of schools (Vermeire et al. 2022), such as the support provided to students, autonomy, and workload, significantly impact the success of the STWT. Additionally, the type of program (vocational vs. general) and the field of study are key determinants (Brunetti and Corsini 2019; Pastore and Zimmermann 2019). Practical work experiences, such as internships and apprenticeships, which students particularly encounter in vocational schools, provide them with valuable on‐the‐job skills while still in education. By establishing strong connections with the labor market, these schools provide significant benefits to students by offering practical opportunities related to their future occupational roles (Bol et al. 2019). Career pathways represent a valuable tool for helping young people select and define clear life projects. In contemporary socioeconomic uncertainty and instability, such pathways promote adaptability and psycho‐social well‐being (e.g., Blustein et al. 2019): when students are well‐prepared, thanks to their educational institutions' resources and guidance, they tend to display greater confidence in their readiness to enter the labor market (Presti et al. 2022).

## 2 STWT in Italy: The Paths for Transversal Skills and Orientation (PCTOs)

The European Union and its Member States are investing considerable efforts and resources to foster the active participation of young people in modern societies characterized by uncertainty and complexity, with the aim of equipping them with the necessary skills for a more effective STWT (OECD 2024a, 2024b). Despite the long‐standing commitment of Italian policies to improving the employability of young people through the enhancement of STWT, the employment rate for recent graduates remains significantly below the EU average (ISTAT Istituto nazionale di statistica 2024). The efforts of the Italian education system to enhance STWT can be systematically traced back to the introduction in 2003 of the “School‐to‐Work Alternance” programs in vocational upper secondary schools (Law No. 53/2003; Legislative Decree No. 77/2005). The School‐to‐Work Alternance (SWA)—replaced in 2019 by the Paths for Transversal Skills and Orientation (PCTOs)—was the first structured teaching method aimed at integrating the development of practical skills into school curricula, strengthening the connections between schools and the labor market. SWA allowed students to “alternate” between classroom learning and workplace training in companies, promoting work cultures among adolescents as an opportunity to engage with diverse cultural practices and experiences related to the world of work (Giancola and Salmieri 2021). Initially not mandatory and aimed only at students in vocational schools, SWA underwent its first major reform in 2015 (Law 107/2015), which made it compulsory for all students in the final 3 years of upper secondary school (grades 11‐13), across all types of schools, including general education schools. This reform reinforced the importance of combining “knowing” with “doing,” thereby strengthening the connection between theory and practical knowledge.

The introduction of PCTOs in 2019 (Decree No. 774/2019) definitively replaced SWA, placing greater emphasis on the holistic development of students. The focus has shifted from the acquisition of practical skills related to specific job sectors towards the development of transversal skills, also known as “soft skills” (e.g., Cinque 2016; Hart et al. 2021; Lucisano, and Rubat Du Merac 2019). This transition was made in line with the broader European recommendations on lifelong learning and career guidance (European Commission 2018), which emphasize the importance of maintaining and acquiring skills that enable young people to fully participate in society and successfully navigate transitions in the labor market. Within this framework, PCTOs placed significant emphasis on equipping adolescents with both practical and transversal skills, which are essential for their future employability regardless of the field of employment, within the perspective of lifelong learning as a guarantee of continued participation in the labor market (Decree No. 774/2019). The introduction of PCTOs reflects the need for a more integrated educational experience that goes beyond the scope of simple work placements, emphasizing holistic skill development with the aim of preparing students to successfully meet the demands of the modern labor market.

To ensure the effective implementation of PCTOs, specific guidelines were issued under Ministerial Decree No. 774 on September 4, 2019. At the application level, these Guidelines include essential elements to draw on to guarantee the effectiveness of these programs.

Firstly, the general recommendation is that a flexible design must be ensured. PCTOs require careful planning, management, and evaluation to be effective. Indeed, a flexible approach is required that can adapt to several factors, including the local context in which the educational institutions operate, the different nature and type of study programs (general or vocational schools), the minimum hours required according to the curriculum, as well as the possibility of employed different and integrated methods, while respecting school autonomy and students' needs.

Secondly, all activities carried out within PCTOs—in organizational and professional settings, in the classroom, in the laboratory, or through simulated experiences—are required to align with the teachings of the student's field of study. Through multiple and varied experiences over time, PCTOs should be structured in phases and activities that provide students with a comprehensive learning experience, supporting their gradual development toward realizing their full potential.

Thirdly, the Guidelines underscore the importance of active student involvement in program planning, encouraging them to reflect on their preferences and expectations. This pedagogical approach aimed to foster students' awareness and encourage them to take direct responsibility for their own learning. In this process, the Guidelines also promote the evaluation and self‐evaluation of these experiences, encouraging meta‐reflection to enhance the PCTOs' “orientation value.” According to the Guidelines, the experience should therefore be documented, and the results shared among students and teachers, including the use of digital tools, to promote the dissemination of best practices.

Finally, to ensure the effective implementation of PCTOs, schools have to designate “internal tutors” from among their teachers. The role of internal tutors is crucial, not only in assisting the school with evaluating host organizations, identifying their educational potential, and addressing any collaboration challenges, but also in providing high‐quality support and fostering strong relationships with students during their PCTO experiences. Tutors monitor activities, address issues, and track and communicate student progress, emphasizing the skills and objectives achieved, encouraging students to evaluate the effectiveness and coherence of their Paths.

Studies conducted so far on the functioning and effectiveness of PCTOs, particularly those involving larger samples, have mainly focused on their governance mechanisms, examining how various stakeholders—such as schools, companies, and local institutions—interact and negotiate choices regarding the design and implementation of the pathways (Giannoni et al. 2024). Additionally, many evaluations have focused primarily on the content of PCTO projects implemented in schools (Fasanella et al. 2021), assessing their role in enhancing students' job opportunities and tertiary education orientation skills, without directly considering students' perspectives or the direct role they play in developing transversal skills. Consequently, there is a gap in the extant literature regarding the actual state of PCTO implementation, which is attributable to the complex nature of these tools. Furthermore, the Guidelines were issued only recently, shortly before the global health crisis linked to the COVID‐19 pandemic, which not only slowed down the implementation process but also its evaluation (Poliandri et al. 2023).

## 3 The Present Study

This study was conducted as part of a broader PRIN 2022 project (Progetto di rilevante interesse nazionale—National Research Project of Significant Interest) [*anonymized for review*], which aims to explore the role of PCTO in developing students' transversal skills and guiding their career choices within the Italian school system, while also investigating the effectiveness of PCTO guideline implementation (Stanzione et al. 2023; Stanzione et al. 2024). Specifically, the PRIN 2022 project focuses on three main areas: analyzing school programs to assess PCTO guideline implementation, conducting mixed‐method case studies on upper secondary school students' PCTO experiences, and surveying university students to evaluate PCTO's influence on career choices.

The current paper presents findings from data gathered during case studies conducted in two technical schools. Although the PRIN 2022 project involves different types of schools (i.e., general and vocational), technical schools (which are part of the vocational schools) were selected for this study, as they were found to have homogeneous study programs (i.e., technical institutes with economics, agricultural, chemistry, and information technology curricula). This allowed us to analyze a sufficient number of participants who shared similar PCTO experiences.

Since PCTO programs are relatively new in Italy, there is a lack of studies evaluating their effectiveness, especially from the students' point of view. This study primarily aimed to fill this gap. To achieve this goal, we investigated the relationship between specific PCTO characteristics and students' transversal skills. Specifically, according to PCTO guidelines, we focused on the following PCTO effectiveness indicators: orientation value, students' design involvement, number of PCTO experiences, and quality of the relationships with the tutor. Then, it was hypothesized that there would be a positive association between PCTO indicators and students' transversal skills (Hypothesis 1). Following the evidence from psychological research in the fields of education and professional development (e.g., Lent 2013), it can be reasonably inferred that the learning experiences and social support inherent to these pathways play a fundamental role in enabling adolescents to manage themselves in multiple areas of life and, as a result, to better adapt to their environment. Furthermore, given that the PCTOs also aim to guide students towards suitable career paths, we hypothesized that the quality of these Paths would be positively associated with the students' career resources (Hypothesis 2), both directly and indirectly, through transversal skills (Hypothesis 3). Indeed, a substantial body of research has demonstrated the significant impact of transversal skills on an individual's career advancement (e.g., Lippman et al. 2015). In addition to being highly sought after in the workplace, transversal skills constitute valuable personal resources that facilitate an individual's adaptation to different aspects of their life (e.g., Majid et al. 2019, 2012). It is important to note that in the absence of a pre‐existing framework in the literature for evaluating the quality of PCTOs, the indicators employed were specifically developed for the present study. Consequently, no a priori hypotheses were formulated regarding the precise associations between these indicators and the specific variables under consideration.

## 4 Methods

### 4.1 Participants and Procedures

This cross‐sectional study involved 567 students (grades 11–13) from two Italian upper secondary schools (technical institutes) in central Italy (M age = 17.30, SD age = 1.02, 72% males). The initial sample consisted of 587 participants. Students who indicated they had not started any PCTO experience were excluded from the sample (*N* = 20). The sample information is shown in Table 1.

**Table 1:** Sample information (*N* = 567).

		School 1 (*N* = 311)		School 2 (*N* = 256)	
		*N*	%	*N*	%
Gender	Male	254	81.70%	154	60.20%
	Female	57	18.30%	102	39.80%
Immigrant background	Native	226	74.30%	245	98.00%
	Second‐generation background	54	17.80%	3	1.20%
	First‐generation background	24	7.90%	2	0.80%
Grade	Grade 11	125	40.20%	97	37.90%
	Grade 12	104	33.40%	88	34.40%
	Grade 13	82	26.40%	71	27.70%
Field of study	Technical and Economic Institute	96	30.90%	43	16.80%
	Technical and Technological Institute	136	43.70%	142	55.50%
	Commercial Technical Institute	37	11.90%	—	—
	Industrial Technical Institute	42	13.50%	41	16.00%
	Agricultural Technical Institute	—	—	30	11.70%

Before the research activities began, informed consent was obtained from the families of minor participants. Additionally, oral assent was required from the minor students, and the research team presented it before proceeding with the questionnaire administration. Adult students indicated their autonomous willingness to participate by signing the informed consent. *The study was approved by the ethics committee of the Sapienza University of Rome*.

### 4.2 Measures

The questionnaire was administered between April and May 2024. The study was conducted via a self‐report questionnaire, which students were invited to complete during school hours using their electronic devices.

*PCTOs indicators*. To assess key elements of PCTOs, identified as effectiveness requirements in the guidelines, an ad hoc tool was developed. Following an analysis of the relevant literature and the PCTO guidelines, the research group constructed a series of questions to enable students to evaluate their PCTO experience. The dimensions to be assessed, as well as the format and type of questions included in the questionnaire, were selected through a collaborative process involving all researchers involved in the project. The final version of the questionnaire was developed through an iterative and inclusive process, in which all inputs were considered, evaluated, and integrated to address the discrepancies identified in the discussion phase. Specifically, the following dimensions were assessed:

1. Orientation value. The orientation value of the PCTO was assessed using three items (*ω* = 0.85) that evaluated both the coherence of the PCTO with respect to the course of study and specific activities within the PCTO, as well as the usefulness of the PCTO for career orientation (response format: 5‐point Likert scale from 1 = not at all to 5 = completely). Specifically, the questions were: *Is your PCTO consistent with your current course of study*? *Did the activities you completed in the PCTO seem consistent with what you were studying*? *Do you think that the PCTO you are attending is useful for your career guidance (job and/or university choices)*?
2. Student Design Involvement. Students' involvement in PCTO design was evaluated using one item (response format: 5‐point Likert scale from 1 = not at all to 5 = completely): *How involved were you in designing your PCTO*?
3. Number of PCTO Experiences. The students were presented with a single item and instructed to indicate the number of distinct PCTO experiences with which they had previously been involved: *How many different PCTO experiences have you had*? By “different experiences” we refer to internships or training sessions carried out in different locations or companies, each involving a distinct set of tasks, schedules, and learning objectives, thus providing students with varied professional and educational experiences.
4. Quality of the relationship with the tutor. Three items (*ω* = 0.87) evaluated the quality of the tutor‐student relationship during students' PCTO experience (response format: 5‐point Likert scale from 1 = not at all to 5 = completely). The area of inquiry was the following: *During your PCTO experience, did you feel supported by the internal PCTO tutor*? *How satisfied are you with your relationship with the school's internal PCTO tutor*? *How useful do you think your internal PCTO tutor at the school was in guiding you along the path*?

*Transversal skills*. Soft Skills Self‐Evaluation Questionnaire (3SQ; Lucisano, and Rubat Du Merac 2019) was used to assess students' perceived transversal skills. 3QS is a self‐evaluation tool, designed for use by secondary school students, which encompasses the following dimensions: trust (3 items; item example: “I am satisfied with myself”), openness (3 items; e.g., “I take other people's opinions into consideration”), collaboration (3 items; e.g., “I willingly participate in group initiatives”), leadership (4 items; e.g., “There are activities in which I guide others”), empathy (3 items; e.g., “I try to put myself in other people's shoes”), commitment (3 items; e.g., “When I carry out an activity, I am determined to complete it”), autonomy (4 items; e.g., “I think independently”), curiosity (3 items; e.g., “I actively seek new information”), problem‐solving (3 items; e.g., “I find as many solutions as possible to the problems I encounter”), and resilience (4 items; e.g., “I know how to deal with crisis situations”). The response format is a 5‐point Likert scale (from 1 = never to 5 = almost always). In the present study, as there were no specific hypotheses regarding the role of PCTOs with respect to each specific transversal skill, a global score was calculated (for a similar use of soft skills scales, see, e.g., Aryani et al. 2021; Feraco et al. 2023). A hierarchical factor analysis revealed that a hierarchical model with a second‐order factor showed a good fit to the data: *X* ^2^_(850)_ = 3860.906, *p* < 0.001, CFI = 0.977, TLI = 0.976, RMSEA = 0.079, SRMR = 0.072. The reliability coefficient of the global measure was adequate (*ω* = 0.96).

*Career Resources*. The Italian version (Pace and Sciotto 2023) of the Career Resources Questionnaire‐Adolescent version (CRQ‐A; Marciniak et al. 2021) evaluated students' career preparedness for successfully transitioning from school to work. Despite the multidimensional nature of this measure, for the aims of this study, we considered only the “motivational resources” dimension because it aligns with the PCTO's career orientation goals. Specifically, motivational resources included the following sub‐dimensions: career involvement (3 items; e.g., “I think work is a very important part of life”), career confidence (3 items; e.g., “I am confident that I will be able to achieve my career goals”), and career clarity (3 items; e.g., “I know what career area I want to aim for”). The response format is a 5‐point Likert scale (from 1 = completely false to 5 = completely true). The results of the confirmatory factor analysis corroborated the presence of three factors, with a general hierarchical factor: *X* ^2^_(24)_ = 41.113, *p* = 0.016, CFI = 0.999, TLI = 0.999, RMSEA = 0.035, SRMR = 0.035. Consequently, a combined score representing the three dimensions was calculated (ω = 0.90). The resulting score indicated the level of motivational resources in career preparedness.

## 5 Data Analysis

Before the main analysis, preliminary descriptive analyses were performed to evaluate missing data and the normality of the distributions. Variables exhibited a low percentage of missing values, ranging from 0.18% to 3.17%. Although the Shapiro–Wilk test for normality was significant (*p* < 0.05), likely due to the large sample size (Razali and Wah 2011), both skewness and kurtosis values were within acceptable ranges, indicating that the data are normally distributed. Following this, a path analysis model was employed to test our hypotheses. The model was estimated using the Full Information Maximum Likelihood estimation method, which accounts for all observed data, including missing data, by maximizing the likelihood function based on the available information (Kline 2023). This approach ensures more accurate parameter estimation by utilizing all the data in the model. Additionally, correlations between endogenous variables were included as free parameters, and bootstrapping was applied to calculate the standard errors.

In our hypothesized model, transversal skills and motivational resources in career preparedness were considered endogenous variables. At the same time, PCTO indicators (Orientation value, Design involvement, Number of PCTO experiences, Quality of the relationship with the tutor) were treated as exogenous variables. Moreover, we included the number of PCTO hours completed by the students at the time of the questionnaire as a covariate. Specifically, students reported an average of 104.53 h of PCTO (SD = 77.34). This considerable variation in the number of hours students attend aligns with PCTO guidelines, which require technical institutes to provide a minimum of 150 h of PCTO over 3 years. As the PCTO Guidelines do not specify a maximum threshold for PCTO hours, the duration of PCTO may vary considerably among students, depending on several factors, such as school organization, local opportunities, and students' needs. For this reason, the perception of the experience could significantly differ between students who, in our sample, reported completing 1 h or those who, for instance, completed 400 h.

The model tested the direct associations of PCTO indicators with transversal skills and motivational resources, as well as the direct associations of transversal skills and motivational resources. Additionally, indirect associations were examined, with transversal skills acting as mediators between PCTO indicators and motivational resources. Model fit was evaluated using various fit indices (e.g., CFI, TLI, RMSEA, SRMR) and the chi‐square statistic (Hu and Bentler 1999). While a nonsignificant chi‐square (*p* > 0.05) indicates good model fit, it is known to be sensitive to large sample sizes, leading to significant values even with minor model‐data discrepancies. Therefore, other fit indices were considered to assess the overall model fit. All analyses were performed using Jamovi 2.4 (The jamovi project 2023), using the Lavaan (Rosseel 2019), SEMLj (Gallucci and Jentschke 2021), and PATHj (Gallucci 2021) packages.

## 6 Results

Correlation results and descriptive statistics are presented in Table 2.

**Table 2:** Descriptive statistics, reliability, and correlations.

	M	SD	Scale range	ω	1	2	3	4	5	6	7
1. Orientation value	2.90	0.88	1–5	0.85	—						
2. Students' design involvement	3.00	0.85	1–5	—	0.44***	—					
3. Number of PCTO experiences	5.70	4.72	1–30	—	−0.14***	−0.07	—				
4. Relationship with the tutor	2.86	0.81	1–5	0.87	0.36***	0.45***	0.07	—			
5. Transversal skills	3.52	0.61	1–5	0.96	0.22***	0.23***	0.08	0.17***	—		
6. Career motivational resources	3.75	0.78	1–5	0.90	0.11**	0.13**	0.10*	0.14**	0.54***	—	
7. Number of PCTO hours	104.53	77.34	1–400	—	0.15***	0.11**	0.15***	0.12**	0.08	0.04	—

*** *p* < 0.001;

** *p* < 0.01;

* *p* < 0.05.

**Figure 1:**
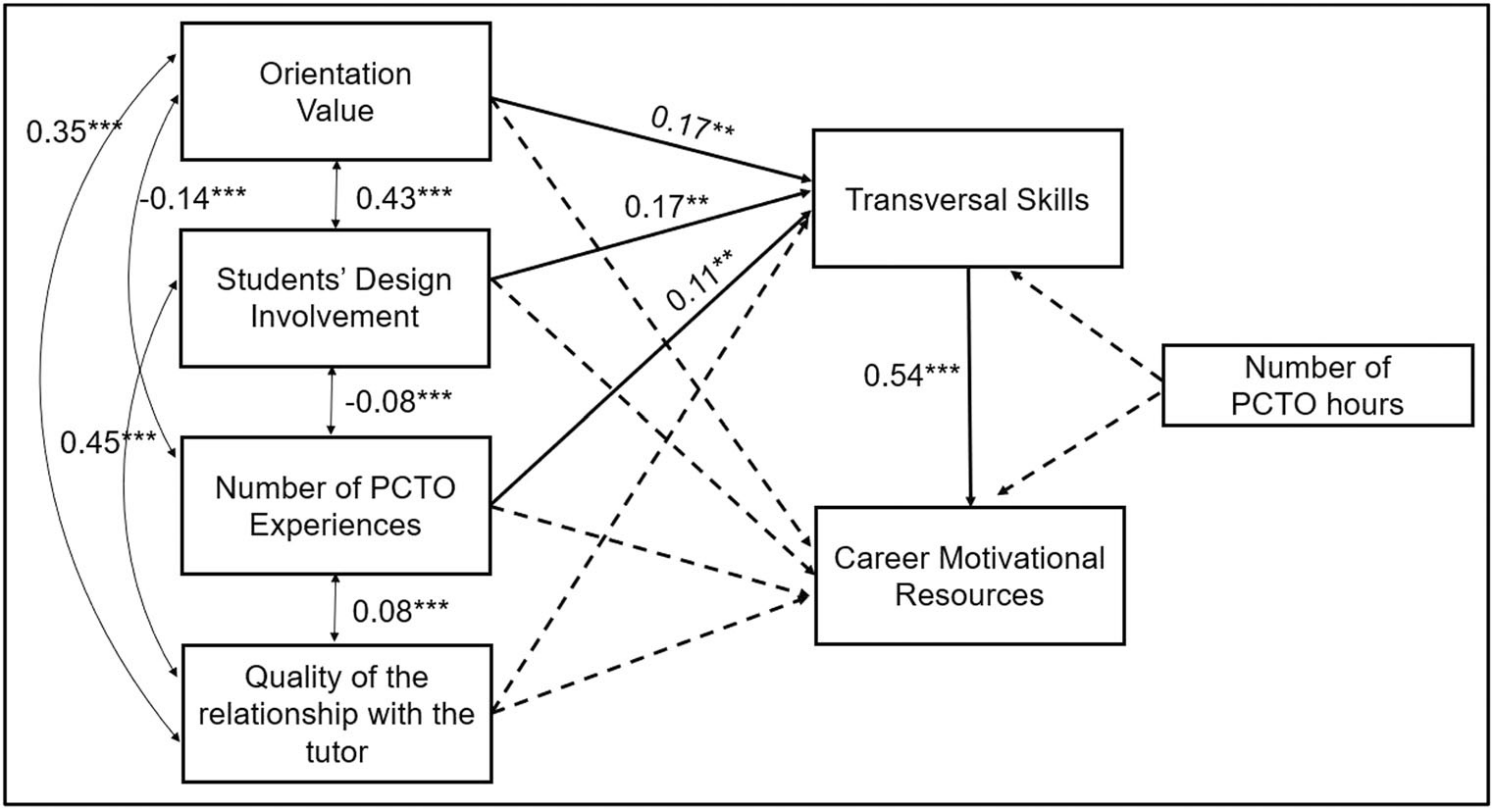
Path analysis model's results. *Note: The model fit indicates a saturated model (CFI and TLI* = *1; RMSEA and SRMR* = 0). All coefficients are standardized. The dotted line indicates a nonsignificant result. ***p < 0.001; **p < *0.01*.

Figure 1 shows the path analysis model results. In line with Hypothesis 1, significant direct associations were found between Orientation value, Design involvement, and Number of PCTO experiences with transversal skills. However, the Quality of the relationship with the tutor did not show a significant direct association. Hypothesis 2 was then not confirmed, as none of the PCTO indicators showed a direct association with motivational resources. However, in line with Hypothesis 3, results showed an indirect association between Orientation value, Design involvement, and Number of PCTO experiences and motivational resources, when mediated by transversal skills (see Table 3). Also in this case, the quality of the relationship with the tutor did not show any significant association.

**Table 3:** Indirect effects.

		*β*	*p*
IE1	Quality of the relationship with the tutor ⇒ Transversal skills ⇒ Motivational resources	0.024	0.343
IE2	Orientation value ⇒ Transversal skills ⇒ Motivational resources	0.090	0.002
IE3	Students' design involvement ⇒ Transversal skills ⇒ Motivational resources	0.088	0.002
IE4	Number of PCTO experiences ⇒ Transversal skills ⇒ Motivational resources	0.058	0.003

Thus, transversal skills not only exhibit a strong direct association with motivational resources but also play a mediating role. Furthermore, the covariate (number of PCTO hours completed) showed no significant association. The model also revealed significant correlations among the exogenous variables (Figure 1). Despite the Number of PCTO experiences having a direct association with transversal skills, the negative correlation between Orientation value and Number of PCTO experiences indicates that the more diverse the PCTO experiences students engage in, the less they perceive these experiences as valuable for career orientation. Ultimately, the model explained 10% of the variance in transversal skills and 30% in career motivation.

## 7 Discussion

The STWT is a pivotal stage in young people's development, particularly concerning their future careers. Beyond the crucial role of imparting invaluable knowledge, educational institutions are responsible for nurturing and developing personal and career resources that equip young individuals with the necessary adaptability to flourish beyond the school context. In this regard, many countries worldwide have adopted structured career guidance pathways. The PCTOs have emerged as a notable example in Italy. PCTOs aim to equip adolescents with the transversal competencies indispensable for future employability, which facilitate, over time and in diverse contexts, the ability to make conscious and fruitful career decisions. Even though these Paths are a structured reality in all Italian secondary schools, there is a lack of studies that evaluate not only the compliance of the PCTOs with the Guidelines but also the psychological processes involved in these experiences.

This study, part of a PRIN 2022 project still in progress (Stanzione et al. 2024), investigated the role of PCTOs in guiding students' career preparedness. Despite the literature on PCTOs providing insight into the challenges and benefits of these pathways, there is a gap in research investigating their efficacy in developing adolescents' transversal skills and influencing their future career and education choices. Our findings, considering students' perspectives, reveal that the alignment of PCTO activities with their field of study, the opportunity for students to engage in multiple and varied experiences over time, and their active participation in PCTO planning contribute to the development of transversal skills, which are increasingly in demand in the labor market (European Commission 2018). These transversal skills emerge as a “key” to guiding adolescents in their future choices, not only to meet the demands of the modern labor market but also to prepare them to participate more actively in the political, economic, and social life as informed and engaged citizens. A further intriguing outcome of this study was that, although tutor support was associated with transversal skills and career resources (see Table 2), when considered alongside the other PCTO dimensions (orientation value, design involvement, number of PCTO experiences) in the general model (see Figure 1), it did not demonstrate significant associations with the outcomes under investigation. This confirms the significant role of the design and orientation of PCTO dimensions and the number of PCTO experiences in students' career adjustment. The results related to the role of tutor in students' PCTO experiences may be attributed to various reasons. First of all, following the organizational structure of PCTO implementation, each class is typically assigned a different tutor. Additionally, PCTO tutors are often teachers who are already burdened with substantial teaching and administrative responsibilities, which limits the time and attention they can devote to the planning and supervision of PCTO activities. As a result, students' experiences with tutors may vary considerably depending on the individual assigned to their class. This heterogeneity, combined with the fact that data were not collected at the class level to preserve anonymity, may have contributed to the lack of significant associations observed in the overall model. Preliminary qualitative analyses (Germani et al. 2025) conducted as part of the same PRIN 2022 project have also highlighted this variability, indicating that the perceived quality and relevance of tutor support are strongly influenced by both contextual and individual factors. However, as widely recognized in the literature (e.g., Hattie 2008), the teacher‐student relationship is a key element for students' overall learning success. Further investigations are therefore needed to understand these results in greater depth. This topic will certainly be an area of further exploration in the qualitative part of the PRIN 2022 project. Additionally, regarding the quality indicators of PCTOs, the negative correlation between Orientation value and Number of PCTO experiences suggested that the more diverse the PCTO experiences students engage in, the less they perceive these experiences as valuable for career orientation. This indicates that while exposure to different work environments helps students develop holistic skills, it may not provide a fully clear understanding of their future career options, as they are exposed to many different experiences. This is an important aspect to consider when assessing the effectiveness of PCTOs and an element that schools must consider for better planning of these pathways.

This study has several limitations that must be considered to better understand its results and design future research. Firstly, due to its cross‐sectional nature, the study cannot establish causality between the observed variables. To gain a deeper insight into the efficacy of PCTOs, future research should focus on longitudinal studies that consider their impact over time. Secondly, it should be noted that the present study involved students attending technical institutes. Future studies should consider additional study programs and compare them with each other, as well as with students who, in other countries, are attending career guidance programs. Furthermore, although the schools selected for this study have similar experiences with PCTOs, information on the specific characteristics of the activities in place has not been investigated. The heterogeneity of PCTO experiences within each institution, and even within individual classrooms, makes it difficult to assess the impact of specific activities on the adaptation indicators considered. Nevertheless, the PRIN 2022 project research group is currently engaged in a series of qualitative studies whose objective is to evaluate these aspects. Additionally, it would also be important to consider the opinions and perceptions of teachers, as these socialization agents play an influential role in determining students' academic and professional success (Marini et al. 2023). Finally, while research has indicated that multiple individual‐ and family‐level factors can influence the career pathways of young people in diverse global contexts (e.g., Marciniak et al. 2022), this study has solely examined contextual predictors of career readiness. Future research must emphasize the factors perpetuating inequalities within the education system, which subsequently impact youth careers.

## 8 Conclusion and Practical Implications

The uncertainty and instability of the current global economic system and labor market have increased policymakers' interest in tools that can support employability and develop skills that are easily transferable across different occupational contexts. In this regard, education systems have strengthened career guidance programs to foster young people's career preparedness, recognizing that effective STWT management enhances the skills, abilities, and competences needed for effective career success.

In line with this perspective, Italy has progressively recognized the strategic relevance of career guidance within its educational policy landscape, aligning with European directives while also implementing national measures to develop context‐specific tools and frameworks (Ministerial Decree No. 774/2019; Ministerial Decree No. 328/2022). PCTOs embody Italy's efforts to implement career guidance tools to facilitate the identification of students' individual aptitudes and support the development of essential competencies for labor market integration. Moreover, by aligning educational paths more closely with students' interests and aspirations, PCTOs aim to contribute to preventing early school dropout and bridge the gap between the skills required by employers and those acquired by students upon completing upper secondary education, thereby enabling a more effective STWT.

The present study, which is part of a broader project of significant national relevance funded by the Ministry of Education, further highlights the interest and commitment of the Italian education system to the issue of career guidance, positioning orientation policies as a central component of students' educational trajectories. Given the institutional relevance of the project and its alignment with Italy's educational priorities (Decree No. 328/2022), the findings are expected to be considered at the national level, contributing to the broader discourse on STWT and the prevention of early school leaving, particularly through the recommendations it will offer for the design, monitoring, and evaluation of PCTOs.

While this paper addresses only one aspect of the main project, it offers some useful insights for improving the effectiveness of PCTO planning in line with national guidelines. As highlighted by the findings, to ensure the effectiveness of PCTOs in guiding students toward informed future choices, it is essential to personalize the pathways and actively involve students in their design. This process requires a systematic analysis of students' needs and interests before the experience, to guide the codesign of the pathways. Schools should collect this information using simple tools, such as questionnaires or reflective sheets, that allow students to express their interests, expectations, and the areas or skills they feel need further development. When students feel involved in the planning of their PCTO experiences, this has been shown to be a key factor in maintaining alignment between the activities and their field of study, thus preserving the orientation value of the pathway. Furthermore, the findings of the present study showed that the relationship between students and their PCTO tutor—a figure to whom the Italian PCTOs and guidance career guidelines attribute a significant role (Decree No. 774/2019; Decree No. 328/2022)—is not associated with the development of soft skills or career motivational resources. However, the quality of the relationship with the tutor was associated with greater student autonomy in making choices about their learning experiences. These findings indicate that tutors have a substantial impact on the efficacy of PCTOs, as they are able to promote effective codesign processes. Consequently, the strategic selection of internal tutors appears to be of paramount importance. To ensure the effective functioning of this process, it is essential to implement a balanced distribution of responsibilities among school staff. This approach is necessary to prevent an excessive workload for teachers, which may hinder their ability to effectively allocate time to the design, monitoring, and evaluation of PCTOs. Furthermore, the extant literature demonstrates that, although teachers involved in school guidance generally manifest positive attitudes toward this role, they express the need for targeted training (Joho et al. 2024). Despite this, one critical issue that is not explicitly addressed in the current Ministry of Education guidelines is the specific training of teachers for this role. This gap will be explored in the upcoming phases of the PRIN project, which aims to investigate and support the professional development needs of PCTO tutors.

Since the PRIN project from which this study is drawn is still ongoing, the indications provided so far regarding ways to improve PCTOs cannot be considered definitive. One of the goals of the PRIN, in fact, is to integrate qualitative and quantitative data to offer a more comprehensive understanding of students' experiences and to identify areas for potential enhancement of PCTOs.

## Author Contributions

Conceptualization: Sara Germani, Mara Marini and Irene Stanzione. Methodology: Sara Germani, Mara Marini and Irene Stanzione. Data analysis: Sara Germani and Mara Marini. Writing – original draft preparation: Sara Germani and Mara Marini. Writing – review and editing: Sara Germani, Mara Marini and Irene Stanzione. Supervision: Irene Stanzione (Principal Investigator).

## Ethics Statement

This study was reviewed and approved by the CERT (Comitato Etico per la Ricerca Transdisciplinare—Ethics Committee for Transdisciplinary Research) of Sapienza University of Rome (protocol number: CERT_18DF411736D).

## Conflicts of Interest

The authors declare no conflicts of interest.
